# Modelling radicalization: how small violent fringe sects develop into large indoctrinated societies

**DOI:** 10.1098/rsos.170678

**Published:** 2017-08-30

**Authors:** Martin B. Short, Scott G. McCalla, Maria R. D'Orsogna

**Affiliations:** 1 Department of Mathematics, Georgia Tech, Atlanta, GA, USA; 2 Department of Mathematical Sciences, Montana State University, Bozeman, MT, USA; 3 Department of Biomathematics, University of California, Los Angeles, Los Angeles, CA, USA; 4 Department of Mathematics, California State University, Northridge, Northridge, CA, USA

**Keywords:** game theory, dynamical systems, modelling social behaviour

## Abstract

We model radicalization in a society consisting of two competing religious, ethnic or political groups. Each of the ‘sects’ is divided into moderate and radical factions, with intra-group transitions occurring either spontaneously or through indoctrination. We also include the possibility of one group violently attacking the other. The intra-group transition rates of one group are modelled to explicitly depend on the actions and characteristics of the other, including violent episodes, effectively coupling the dynamics of the two sects. We use a game theoretic framework and assume that radical factions may tune ‘strategic’ parameters to optimize given utility functions aimed at maximizing their ranks while minimizing the damage inflicted by their rivals. Constraints include limited overall resources that must be optimally allocated between indoctrination and external attacks on the other group. Various scenarios are considered, from symmetric sects whose behaviours mirror each other, to totally asymmetric ones where one sect may have a larger population or a superior resource availability. We discuss under what conditions sects preferentially employ indoctrination or violence, and how allowing sects to readjust their strategies allows for small, violent sects to grow into large, indoctrinated communities.

## 1. Introduction

The past few decades have seen a remarkable change in the motivations leading to armed conflicts worldwide, in how and by whom populations are being mobilized, and in the ways violence unfolds in societies [1]. The character of present warfare is increasingly civil, internal and sectarian as opposed to the more international, external wars fought until the mid-twentieth century. The latter were mostly driven by economic, imperialistic and expansionistic intents—such as the two world wars—or by nationalistic and ideological motives—such as the quest for independence of former colonies, or the attempt to forcefully establish capitalism over communism. Contemporary conflicts on the other hand are marked by ethnic, cultural or religious undertones that were not a source of full fledged rivalry or hostility between groups until triggered by specific political or military events. Examples include the Balkan and Rwandan civil wars, the secessionist conflict in Chechnya, the Darfur war, the Lebanese civil war and religious conflicts in Northern Ireland. In more recent years, the Middle East has experienced a dramatic surge in tension between the Sunni and Shia denominations of Islam, particularly in Iraq and Syria [2,3] (table 1).

**Table 1.** Parameter list for sects *i*=A,B.

*N*_*i*_	total faction population
λ_*i*_	spontaneous radicalization rate
*μ*_*i*_	spontaneous de-radicalization rate
*p*_*i*_	indoctrination rate
*k*_*i*_	attack rate
*ω*_*i*_	maximum radical activity rate

While each of these past or present conflicts must be placed in its own historical context and carries its unique *raison d’$\hat{e}$tre*, a few common threads can be found. Most arise as small, local conflicts, often building on denominational or cultural divisions that had been simmering for years but that had been reined in by a legitimate or de facto authority: occupying forces, the government or a dictator. The collapse or fall of the latter, especially in societies with less concern for human rights, has often led to chaotic free-for-all states, marked by widespread abuse, violence against civilians and terror. Dormant identity divisions have resurged as a need and a way to protect one’s own. Emerging sectarian groups are often led by fanatics, rebels or irregular militiamen that, within a failed nation state, are able to expand their reach and influence without restraint, such as in the case of the Islamic State [4]. Divisions and hostility are often deepened by the continued use of propaganda and identity arguments, whether constructed or factual, to recruit militants and sympathizers and to present the ‘other’ as an enemy or a scapegoat. Against this backdrop, and often exacerbated by poverty and lack of education and opportunity, the radicalization of communities drives individuals that had once lived side by side in relative harmony towards segregation and to the ever increasing glorification of violence.

Being able to understand how individuals and societies become radicalized [5–7] is an important step in trying to prevent extremism in volatile, unstable societies that are not yet completely polarized, and for the development of possible, effective anti-radicalization strategies in those that are. The goal of this paper is to develop a mathematical model to study the radicalization of sectarian communities. We aim to incorporate several sociologically relevant factors: active indoctrination by established radicals, psychological responses to attacks and possible de-radicalization efforts.

Within the general context of political or religious radicalization, previous population models grouped individuals depending on level of zeal [8,9], allowing individuals to become more or less fanatic depending on their interactions with others. Network models [10–13] and applications to specific cases such as the radicalization of Basques by the ETA terrorist organization [14] have also been studied. Models targeted to specifically describe the Middle East include the study of public support towards occupying forces in Iraq via population-based [15] or lattice-type models [16,17] where sites are populated with civilians and insurgent agents in proportions derived from ethnic maps of Baghdad [18]. Attacks, migrations and safety issues are included, as well as policing strategies to mitigate violence.

In our work, we consider two opposing populations, each containing a more and a less radical component that respond to and/or cause attacks and/or disseminates propaganda. As we shall describe in the next section, the model we present has a more dynamic and adversarial character than the mostly ‘opinion’ models introduced so far, as we include the possibility of moderates radicalizing not only in response to internal indoctrination, but also in direct response to the offensive actions carried out by the opposing party. Indeed, we allow the radical component of one group to take advantage of the actions of the other for their own proselytizing efforts. Several parameters will be introduced, for example, to describe the willingness of one faction to carry out attacks on the other, or the degree of de-radicalization. Each of these parameters will embody given tactical possibilities or societal reactions, so that different regimes can be associated with different types of sects and antagonism between them. Parameters can also change due to the intervention of policing forces, the international community or pacifying groups, so that our equations can describe a variety of situations. We use utility functions that radicals seek to optimize by selecting relevant ‘strategies’ as they interact with each other. Using game-theoretic ideas, we study how societal dynamics unfold depending on the choices made. The insights gained by our work may be useful in understanding why radical groups behave as they do, why certain archetypal rivalries persist and may help identify ways to disrupt mounting cycles of violence.

## 2. The model

For concreteness, we frame our model in terms of religious extremism, although the discussion that follows can be readily applied to any political or nationalistic ideology that causes ‘violent’ rivalries. We consider a total population of individuals that can adhere to belief A or B, and be radical or moderate within each group. Here, radicals are defined by their willingness to ‘attack’ members of the opposite group, via direct physical violence, the dissemination of incendiary comments or the desecration of religious symbols, for example. At time *t*, the population is thus divided into *r*_A_(*t*) radicals of faith A, *r*_B_(*t*) radicals of faith B, *n*_A_(*t*) moderates of faith A and *n*_B_(*t*) moderates of faith B. We also assume the total population for factions A and B to be set at *N*_A_ and *N*_B_, respectively, so that *r*_A_(*t*)+*n*_A_(*t*)=*N*_A_ and *r*_B_(*t*)+*n*_B_(*t*)=*N*_B_. These conditions can be relaxed, but we use them as a starting point. The entire population is denoted by *N*=*N*_A_+*N*_B_. Given the fixed sect population assumption, we only need to study the dynamics of the *r*_A_ and *r*_B_ radicals.

Let us focus on sect A, and consider radicalization and de-radicalization within this sect. Our first assumption is that these processes are modulated by a collective ‘sensitivity’ parameter *s*_A_: as *s*_A_ increases so does radicalization, while de-radicalization decreases. This quantity may be viewed as a state of alertness, or as the propensity of members of sect A to radicalize; for example, *s*_A_ will be low in situations of calm but may increase under hostilities perpetrated by members of sect B.

Radicalization will take two forms: either as a spontaneous process or after personal discussions, social media or mass indoctrination campaigns that expose an individual to already radicalized ones. Such pathways to radicalization are well established and have been reported in the sociology, psychology and political science literature [7,19–22]. On the other hand, de-radicalization is assumed to occur as an internal process only: we assume there are no active attempts by non-radicals to change the minds of the radicals. Social science studies report that de-radicalization may be due to ideological disengagement stemming from the perception of lesser threats, ageing out, or possible exit programmes crafted by non-governmental or international organizations [23,24]. Since age-dependency or third-party interventions are outside the scope of this work, for simplicity we assume de-radicalization to depend solely on the sensitivity level of the population. We can thus write an expression for the time evolution of the number of radicals *r*_A_ as follows:

2.1 $${\dot{r}}_{A}=n_{A}\left[\lambda_{A}s_{A}+p_{A}s_{A}\frac{r_{A}}{N_{A}}\right]-\mu_{A}f(s_{A})r_{A}.$$

The first term on the right-hand side of equation (2.1) represents spontaneous radicalization described by the intrinsic rate λ_A_ and modulated by the sensitivity parameter *s*_A_. The second term represents radicalization in response to indoctrination from the *r*_A_/*N*_A_ fraction of active radicals at rate *p*_A_, and similarly modulated by *s*_A_. Finally, we assume de-radicalization occurs at an intrinsic rate *μ*_A_ modulated by a sensitivity-dependent function *f*(*s*_A_) that decreases with *s*_A_ so that a highly sensitive population is more unlikely to de-radicalize. The dynamics of the *r*_B_(*t*) radicals are described in the same way, with all labels A replaced with B in equation (2.1), and where rates λ_B_,*p*_B_,*μ*_B_ and the population *N*_B_, may differ from their A counterparts. Our complete model thus consists of two coupled equations for *r*_A_,*r*_B_ where the two factions may or may not be symmetrically balanced in their strengths and responses.

Note that we have not yet specified the form of *s*_A_. If this quantity is selected to be a numerical value independent of the dynamics of sect B, the two factions are effectively uncoupled and the dynamics of *r*_A_(*t*) are completely determined by equation (2.1) once the initial condition and the form for *f*(*s*_A_) are set. Interactions arise only when the sensitivity of sect A depends on the actions of sect B members and/or vice versa. Within the scenario of a functional dependence of *s*_A_ on *r*_B_ and/or of *s*_B_ on *r*_A_, we assume that members of a given sect adjust their responses to indoctrination and their spontaneous radicalization and de-radicalization depending on the actions taken by the other sect. For example, we can model ‘attacks’ from one sect to the other and assume that the sensitivity of the victim sect will depend on the intensity or frequency of these attacks. In the next sections we will look first at the simple case of non-interacting sects, where *s*_A_ is a numerical value independent of sect B, and the more complex case of interacting sects, where the notion of attacks will be quantified and functional forms for *s*_A_(*r*_B_) and *s*_B_(*r*_A_) will be introduced. We will determine steady states, stability and bifurcations. Furthermore, we will identify parameters that can be adjusted either by radicals or by external forces, such as the intensity of indoctrination or the frequency of attacks, and study how changes in these parameters affect the dynamics and societal outcomes through the aid of objective functions to be optimized.

## 3. Non-interacting sects

As a starting point and benchmark for more complex scenarios, we first consider non-interacting sects where *s*_A_ is a constant on [0,1]. Here, the radicalization of sect A individuals does not depend on the dynamics or actions of sect B members. This is plausible if the latter are incapacitated or choose not to engage with sect A members. For mathematical simplicity we set

3.1 $$f(s_{A})=1-s_{A}.$$

Under these assumptions, the model in equation (2.1) reduces to a simple ODE for *r*_A_:

3.2 $${\dot{r}}_{A}=(N_{A}-r_{A})\left[\lambda_{A}s_{A}+p_{A}s_{A}\frac{r_{A}}{N_{A}}\right]-\mu_{A}(1-s_{A})r_{A}.$$

Note that the choice *s*_A_=0 leads to a vanishing radical population $r_{A}(t\to \infty )=0$, while setting *s*_A_=1 leads to a society saturated with radicals $r_{A}(t\to \infty )=N_{A}$ regardless of initial conditions, assuming λ_A_ and *μ*_A_ are non-zero; we will assume λ_A_,*μ*_A_>0 from here onward, unless specifically noted otherwise. For 0<*s*_A_<1, we non-dimensionalize equation (3.2) by rescaling time via *t*′≡*μ*_A_(1−*s*_A_)*t* and by considering fractional populations so that *r*_A_′*N*_A_≡*r*_A_ and *n*_A_′*N*_A_≡*n*_A_. We also redefine *p*_A_′≡*p*_A_*s*_A_/*μ*_A_(1−*s*_A_),λ_A_′≡λ_A_*s*_A_/*μ*_A_(1−*s*_A_). Using these substitutions and dropping prime indices and sect labels for simplicity, equation (3.2) can be rewritten as:

3.3 $$\dot{r}=-pr^{2}+gr+\lambda ,$$

where

3.4 g=p−λ−1.

The above equation can be solved exactly, as it is in the form of a Riccati equation. Specifically, if at *t*=0 there are *r*(0)=*δ* radicals, equation (3.3) has solution

3.5 $$r(t)=\frac{r_{1}(\delta -r_{2})-r_{2}(\delta -r_{1})\;e^{-p(r_{1}-r_{2})t}}{(\delta -r_{2})-(\delta -r_{1})\;e^{-p(r_{1}-r_{2})t}},$$

where

3.6 $$r_{1,2}=\frac{g\pm \sqrt{g^{2}+4\lambda p}}{2p}$$

and the labels 1,2 apply to the plus and minus signs, respectively. Note that, in order to be physically acceptable, the above solutions must satisfy the constraint 0≤*r*≤1, since the renormalized *r* is now a fractional population. It can be shown that for λ,*p*>0, *r*_1,2_ are such that 0<*r*_1_<1 and *r*_2_<0. Since *r*_1_>*r*_2_, at steady state the exponential terms in equation (3.5) vanish and $r(t\to \infty )=r_{1}$, independently of the initial condition *δ*. We thus find that a radical population will always emerge, even if *δ*=0, as long as λ,*p*>0. For *p*=0, at steady state r(t→∞)=λ/(λ+1), while for λ=0, $r(t\to \infty )=r_{1}=1-1/p$ if *p*>1, else r(t→∞)=0. Therefore, the only case where no radicalization occurs is for λ=0,*p*≤1. In dimensional units, this is λ=0, and *ps*<*μ*(1−*s*); that is, radicals will always emerge unless there is no spontaneous radicalization and de-radicalization is always stronger than radical persuasion. In this case, the presence of radicals is short-lived at best. For all other dimensional parameters λ,*p*,*μ*, radical groups will emerge and persist. Furthermore, since *r*_1_ increases with *p*, the number of radicals at steady state will also increase with *p*. Hence, for non interacting sects, if radicals aim to increase their ranks, the best ‘strategy’ is to choose the largest indoctrination rate *p* possible.

## 4. Interacting sects

We now consider the case where sects interact with each other through interdependent sensitivity functions *s*_A_(*t*) and *s*_B_(*t*). As described above, we assume that the propensity of sect A members to radicalize depends on acts of aggression coming from sect B, such as military raids, the dissemination of incendiary materials, or the desecration of religious symbols. We assume these ‘attacks’ are performed or instigated solely by the *r*_B_(*t*) radicals within sect B. Our basic modelling assumption is that the higher the incidence of these aggressions from the opposite faction, the higher the propensity for individuals to self-radicalize and to respond to internal fanatic propaganda. In this context, sensitivity is an ‘external’ drive, as it modulates radicalization within a group in response to the actions or characteristics of the other.

To formalize our mathematical definition of *s*_A_(*t*), we introduce *k*_B_, the rate of radical attacks against sect A performed by each radical member of sect B, so that *s*_A_(*t*) is proportional to *k*_B_*r*_B_(*t*). We assume that due to finite preparation times, logistic difficulties, and/or limited resources, *k*_B_ cannot exceed a maximum threshold *ω*_B_. Correspondingly, sect A radicals attack sect B members at rate *k*_A_ per radical with an upper limit set at *ω*_A_. To complete our definition of *s*_A_(*t*), we posit that the effects on *s*_A_(*t*) of attacks by sect B are mitigated by the attack capabilities of sect A itself, expressed as the entire population *N*_A_ being mobilized and counter-attacking at maximal rate *ω*_A_. We thus assume *s*_A_(*t*) is also inversely proportional to *ω*_A_*N*_A_ and write

4.1 $$s_{A}(t)\equiv \frac{k_{B}r_{B}(t)}{\omega_{A}N_{A}}=\frac{\omega_{B}N_{B}}{\omega_{A}N_{A}}\frac{k_{B}r_{B}(t)}{\omega_{B}N_{B}}\equiv x\frac{k_{B}r_{B}(t)}{\omega_{B}N_{B}}.$$

As expressed by equation (4.1), the externally driven sensitivity *s*_A_(*t*) is given as the ratio between the number of attacks per unit time the *r*_B_(*t*) radicals actually impart on sect A members, and the hypothetical maximal attack rate members of sect A could inflict on members of sect B in retaliation or in defence. In this context, sensitivity is a measure of the relative aggression capabilities of the two groups. The last product on the right-hand side of equation (4.1) recasts *s*_A_(*t*) in a slightly different manner: the dimensionless term *k*_B_*r*_B_(*t*)/*ω*_B_*N*_B_ is the ratio between the total attack rate of sect B members and their *own* maximal aggression rate given by *ω*_B_*N*_B_. It is a term controlled solely by the dynamics and parameters of members of sect B. The dimensionless quantity *x*≡*ω*_B_*N*_B_/*ω*_A_*N*_A_ measures the aggressive capability of sect B per unit time with respect to sect A. It is the ratio between the respective maximal aggression rates of the two sects if attacks were carried out by their entire populations. This representation of *s*_A_(*t*) will prove to be useful in analysing our model and will provide a better interpretative context in the following sections.

So far the model contains several rates to describe the actions taken per unit time by members of a given sect: for sect A these are λ_A_, *μ*_A_, *p*_A_ and *k*_A_, representing, respectively, the intrinsic radicalization and de-radicalization rates and the indoctrination and attack rates; similarly for sect B. We now assume that the first two, λ_A_ and *μ*_A_, are inherent to sect A, and cannot be changed at will either by radicals or non-radicals. At most, they can be modulated by the sensitivity as described earlier. On the other hand, we assume that radicals can select their attack and indoctrination rates *k*_A_ and *p*_A_ to optimize their intent, which may be, for example, to maximize the number of radicals or to inflict the highest damage to members of the opposite sect. Resource constraints, however, pose a limit on these activities, so that if indoctrination is favoured, attacking becomes less of a priority and vice versa. In this respect *ω*_A_ represents the total amount of resources available per unit time to radicals of sect A to carry out their activities. We thus pose

4.2 $$k_{A}+p_{A}\leq \omega_{A},$$

so that the maximal attack rate *k*_A_=*ω*_A_ can be attained only if there is no active indoctrination and *p*_A_=0. In principle, equation (4.2) could include a relative weight to measure how resource expenditure for indoctrination compares to that for attack activities. This would allow us to explore cases where indoctrination may be more or less resource intensive than attacking the opposite sect. However, it can be shown that once introduced, this relative weight can be subsumed in the non-dimensional parametrization that we illustrate later, so we omit it for simplicity. We shall refer to the combination of (*p*_A_,*k*_A_) values under the constraint in equation (4.2) as the ‘strategy’ of sect A, and similarly for sect B.

The definition for *s*_B_(*t*) is the same as equation (4.1) with the A and B subscripts switched, so that

4.3 $$s_{B}(t)\equiv \frac{k_{A}r_{A}(t)}{\omega_{B}N_{B}}=\frac{1}{x}\frac{k_{A}r_{A}(t)}{\omega_{A}N_{A}}.$$

For simplicity and without loss of generality, we will later assume that sect A has a maximal aggression rate *ω*_A_*N*_A_ that is at least as large as that of sect B, so that

4.4 $$x=\frac{\omega_{B}N_{B}}{\omega_{A}N_{A}}\leq 1.$$

This assumption also implies that the stronger sect A is inherently less sensitive to radicalization than the weaker sect B [25,26]. Indeed, equations (4.1), (4.3) and (4.4) imply that 0≤*s*_A_≤*x* while 0≤*s*_B_≤1/*x*. Similarly, we assume that *k*_B_ obeys the analogue to equation (4.2), where all A subscripts are switched to B. We complete our model by proposing a simple form for *f*(*s*_*i*_), for *i*=A,B,

4.5 $$f(s_{i})=1-xs_{i},$$

which allows *f* to be decreasing and non-negative, since *xs*_*i*_≤1 for *i*=A,B. Our model is now completely specified by equations (2.1), (4.1) and (4.5), and the corresponding equations for sect B, which we now non-dimensionalize for simplicity. Similarly to above, we define *r*′_*i*_=*r*_*i*_/*N*_*i*_ for both sects *i* = A,B and rescale *t* by the de-radicalization time scale of sect A so that *t*′=*μ*_A_*t*. We also define *k*′_*i*_=*k*_*i*_/*ω*_*i*_, λ′_*i*_=λ_*i*_/*μ*_A_, *p*′_*i*_=*p*_*i*_/*μ*_A_, *ω*′_*i*_=*ω*_*i*_/*μ*_A_, for *i*=A,B and *μ*′_B_=*μ*_B_/*μ*_A_. Using these definitions, and dropping the prime symbol for simplicity, we find

4.6 $${\dot{r}}_{A}=xk_{B}r_{B}(1-r_{A})(\lambda_{A}+p_{A}r_{A})-r_{A}+x^{2}k_{B}r_{A}r_{B}$$

and

4.7 $${\dot{r}}_{B}=\frac{k_{A}r_{A}}{x}(1-r_{B})(\lambda_{B}+p_{B}r_{B})-\mu_{B}r_{B}+\mu_{B}k_{A}r_{B}r_{A},$$

coupled with the dimensionless resource constraint

4.8 $$k_{i}+\frac{p_{i}}{\omega_{i}}\leq 1\;\text{for }i=\text{A,B}.$$

We will refer to equation (4.8) with the strict equal sign as the constraint line.

### 4.1. Fully symmetric, non-constrained sects

We first consider the case of two completely symmetric sects where all parameters and initial conditions for the two factions coincide, such that *r*_A_(*t*)=*r*_B_(*t*)=*r*(*t*) for all *t* and *x*=1. We can drop all sect subscripts from our notation in this case and write

4.9 $$\dot{r}=-pkr(r^{2}-Br+C),$$

where

4.10 $$B=\frac{p-\lambda +1}{p}\;\mathrm{and}\;C=\frac{1-\lambda k}{pk}.$$

We first analyse these equations without the constraint in equation (4.8); later we will introduce the constraint and determine regions of validity. Equation (4.9) allows for the ‘pacified’ solution *r*=0, where no radicals arise in either sect. Alternatively, radical populations will be described by the roots of the quadratic term in equation (4.9), where solutions must satisfy the condition 0≤*r*≤1, as *r* is a fractional population. Given an initial radicalization *r*(*t*=0)=*δ*, exact solutions are given by

4.11 $$r^{\Delta_{12}}\frac{{\left(r-r_{1}\right)}^{r_{2}}}{{\left(r-r_{2}\right)}^{r_{1}}}=\delta^{\Delta_{12}}\frac{{\left(\delta -r_{1}\right)}^{r_{2}}}{{\left(\delta -r_{2}\right)}^{r_{1}}}e^{-pkCt\Delta_{12}},$$

where

4.12 $$\begin{array}{r} r_{1,2}=\frac{B\pm \sqrt{B^{2}-4C}}{2}, \end{array}$$

4.13 $$\begin{array}{r} \Delta_{12}\equiv r_{1}-r_{2}=\sqrt{B^{2}-4C}. \end{array}$$

We have labelled *r*_1,2_ so that the plus sign corresponds to *r*_1_ and the minus sign to *r*_2_. Together with *r*=0, *r*=*r*_1,2_ are the zeros of the right-hand side of equation (4.9), representing possible steady states of the system. Instead of analysing equation (4.11), we consider the stability of the possible steady state solutions arising from equation (4.9). In particular, whether the *r*=0,*r*=*r*_1,2_ solutions are physical and/or stable at steady state depends on the values of the chosen parameters. A straightforward analysis allows us to distinguish four regimes in terms of *k* and *p*. Let

4.14 $$K(p)=\frac{4p}{p^{2}+{\left(1-\lambda \right)}^{2}+2p(1+\lambda )}.$$

We find

— If k<K(p), then *r*_1,2_ are complex and unphysical, leaving *r*=0 as the only steady state. Here, sects are not aggressive enough to entice a stable radical population. Radicalization efforts fail and at equilibrium the unique stable state is *r*=0.
— If K(p)≤k<1/λ and *p*<|1−λ| then *r*_1,2_ are real, but are not in the range of physically allowed values. Here, attacks between sects are vigorous, enhancing both spontaneous and persuasive radicalization on both sides, and decreasing de-radicalization. However, attacks alone are not sufficient to maintain radicalization, and indoctrination levels are too low to compensate. At steady state, *r*=0 for all initial conditions.
— If K(p)≤k<1/λ and *p*≥|1−λ| then *r*_1,2_ are real, and are both in the range of physically allowed values. Attacks between sects are vigorous, enhancing both spontaneous and persuasive radicalization on both sides, and indoctrination is significant. De-radicalization efforts may or may not be enough to halt the process and the presence of radical factions at steady state will depend on initial conditions. For small enough radical factions 0≤*δ*<*r*_2_, radicalization is not sustainable as t→∞ and $r(t\to \infty )=r^{\mathrm{eq}}=0$. A finite steady state radical population will arise at $r(t\to \infty )=r^{\mathrm{eq}}=r_{1}$ only if initial radicalization is large enough, with *r*_2_<*δ*≤1.
— If *k*≥1/λ, both *r*_1,2_ roots are real with *r*_2_<0 and 0<*r*_1_≤1. Sects are aggressively attacking each other and are highly prone to radicalization, so that regardless of the indoctrination activity *p* of the already radicalized fraction, a radical population will emerge and persist. Note that if we impose *k*≤1, this scenario can arise only if λ≥1, which in dimensional form is λ≥*μ*; i.e. the intrinsic rate of spontaneous radicalization must be higher than that of de-radicalization. The unique stable state for the fraction of radicals is $r(t\to \infty )=r^{\mathrm{eq}}=r_{1}$ for all initial conditions.

The same behavioural regimes can be identified by working directly with equation (4.11) and by considering different long time trends as a function of *C*,*r*_1,2_. We can thus conclude that an active radical population will persist for t→∞ if the radicals are either (i) very violent, in which case indoctrination levels are immaterial; or (ii) moderately violent while employing enough propaganda. All other conditions lead to no radical populations at steady state.

### 4.2. Fully symmetric, constrained sects

**Figure 1.**
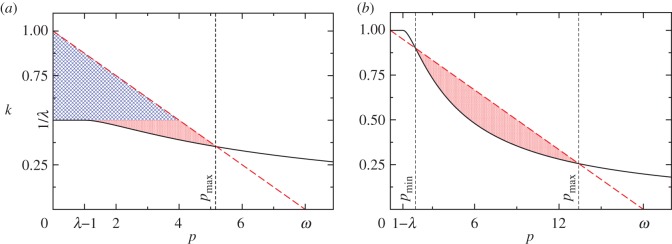
General feasible strategies (*p*,*k*) (shaded regions) that lead to sustained radical populations for (*a*) λ>1 and (*b*) λ<1. Blue shading represents regions where any initial condition will lead to sustained radicalization; red shading indicates regions where only large enough initial radical populations lead to sustained radicalization. In both panels, the dashed red line is the constraint line defined by equation (4.8), while the solid black curve is *K*(*p*) for *p*≥|1−λ| and the lesser of 1 and 1/λ for *p*<|1−λ|. Note that in scenario (*a*), there are always feasible strategies that lead to a non-zero radical population, namely those in which *k*>1/λ, regardless of *ω*. In scenario (*b*), feasible strategies arise only if *ω* is sufficiently large for a given λ.

We now consider the two fully symmetric sects being subjected to limited resources and analyse the above results under the (*p*,*k*) constraint in equation (4.8). We illustrate our results in figure 1 and consider two cases: λ>1 in figure 1*a* and λ≤1 in figure 1*b*. In both cases, the constraint line *k*=1−*p*/*ω* given by equation (4.8) is depicted as a red dashed line, while the solid black curve is the function k=K(p) given by equation (4.14) for *p*≥|1−λ| and min(1,1/λ) for *p*<|1−λ|.

For λ>1, the curve k=K(p) attains its maximum at (λ−1,1/λ), and the black curve of figure 1*a* intersects the constraint line *k*=1−*p*/*ω* only once, at *p*=*p*_max_. For *ω*≥λ this intersection occurs at *p*_max_≥λ−1, vice versa for *ω*<λ at *p*_max_<λ−1. In figure 1*a*, we assume *ω*>λ. We can now use the results from §4.1 to determine behaviours in (*p*,*k*) space subject to the constraint. For 1/λ≤*k*≤1−*p*/*ω* radical groups will emerge at steady state, regardless of initial conditions. This region is blue-shaded in figure 1*a*. For K(p)≤k≤min(1/λ,1−p/ω), radical groups will also persist at steady state, but only if initial conditions are chosen so that *r*(*t*=0)=*δ* with *r*_2_<*δ*≤1 with *r*_2_ defined in equation (3.6). This region is red-shaded in figure 1*a* and exists only if the k=K(p) curve and the *k*=1−*p*/*ω* constraint intersect for values of *p*_max_≥λ−1, that is for *ω*>λ as shown in figure 1*a*. Thus, if the initial radical population is large enough, a sustained radical population will arise at steady state for all values of 0≤*p*≤*p*_max_, as depicted by the union of the blue- and red-shaded areas in figure 1*a*. Our analysis leads to the conclusion that for symmetric sects characterized by large intrinsic radicalization rates λ and subject to the constraint in equation (4.8), non-zero radical populations may persist at steady state if *p*≤*p*_max_ and reciprocal attacks as determined by *k* are large enough. For *k*>1/λ and *p*<*ω*(1−*k*), radical sects will emerge regardless of initial conditions; for *k*≤1/λ, only if initial conditions are favourable. If indoctrination rates *p*>*p*_max_ are too large at the expense of *k*, no sustained levels of radicals may emerge.

For λ≤1, the curve k=K(p) attains its maximum at (1−λ,1). The black curve in figure 1*b* defined as K(p) for *p*≥1−λ and as min(1,1/λ) for *p*<1−λ, will intersect the non-dimensional constraint line *k*=1−*p*/*ω* at (*p*,*k*)=(0,1). However, this intersection will lead to vanishing radical populations at steady state, as discussed in the previous sub-section. For values of *p*>1−λ the curve k=K(p) will cross the constraint line *k*=1−*p*/*ω* either zero, one or two times, depending on the magnitude of *ω* for a given λ. If there are no intersections, following the results derived in the previous sub-section, no radical populations will persist at steady state, regardless of initial conditions, since k<K(p) for all values of (*p*,*k*) subject to the constraint in equation (4.8). If there are two intersections, which we label (*p*_min_,*k*_min_) and (*p*_max_,*k*_max_), all values of (*p*,*k*) chosen between them such that K(p)≤k≤1−p/ω lead to finite radical populations at steady state, but only under the proper initial conditions. This scenario is shown in figure 1*b*, where the red-shaded region contains all (*p*,*k*) values associated with the emergence of two intersections. The transition between the zero intersection and two intersection cases occurs when the chosen *ω* and λ values lead to a single intersection between k=K(p) and *k*=1−*p*/*ω*. To find these values we impose K(p)=1−p/ω and $K^{\prime }(p)=-1/\omega$. These equations yield a critical value *ω*_*c*_(λ) such that for *ω*≥*ω*_*c*_(λ) two intersections may be found, while for *ω*<*ω*_*c*_(λ) none arise. Importantly, *ω*_*c*_(λ) is an increasing function of λ, and *ω*_*c*_(0)=4 while $\omega_{c}(1)=(11+5\sqrt{5})/2$. Thus, if *ω*<4, there will never be any intersections between the constraint line and k=K(p) for any λ<1 and no radical population can be sustained at steady state. On the other hand, values of $\omega \geq (11+5\sqrt{5})/2$ will result in two intersections for any λ<1 so that long-term radical populations may persist given a large enough initial radical population and proper (*p*,*k*) choices. We can thus conclude that for moderate self-radicalization rates λ, a large radical population will be sustained only if enough resources *ω* are available, if there is an initial, nucleating group of enough radicals, and if indoctrination and attack rates are judiciously balanced.

We now turn to identifying the *optimal* strategy (*p*,*k*) that radical groups should adopt. So far, we have tacitly assumed that the goal of the two radical factions is to simply perpetuate their existence at steady state so that any (*p*,*k*) points within the shaded regions of figure 1*a* and *b* could be employed, conditioned on starting with the proper initial conditions. We denote those shaded areas as feasible sets. However, radical groups may have much more nuanced goals. Let us now introduce an objective function that radical populations seek to optimize, by identifying the *best* (*p*,*k*) values. We propose a relatively simple objective function, given in dimensionless parameters for radicals in sect A by

4.15 $$U_{A}(p_{A},k_{A};p_{B},k_{B})=r_{A}^{\mathrm{eq}}-\frac{N_{B}}{N_{A}}k_{B}r_{B}^{\mathrm{eq}}.$$

Similarly, *U*_B_(*p*_B_,*k*_B_;*p*_A_,*k*_A_) for radicals of sect B is given by exchanging the labels A and B in equation (4.15). The above choice for *U*_A_ implies that radicals seek to expand their ranks, through the *r*^eq^_*A*_ term, while minimizing attacks from their rival group, represented by $k_{B}r_{B}^{\mathrm{eq}}$ and modulated by the ratio of their respective populations *N*_B_/*N*_A_. For example, if sect B has a larger population that sect A, the objective of sect A radicals is skewed towards preventing attacks, while if sect B has a lower population that sect A, the objective of the sect A radicals is biased toward maximizing radicalization in sect A. Because of the interdependent sect dynamics, the two terms in equation (4.15) are related to each other in a non-trivial way. For example, an increase in the population of sect A radicals will be accompanied by an increase in the number of attacks against sect B members. This will in turn spur the growth of a larger radical population within sect B, who will now engage in more hostile activities against sect A. Of course, this is counter to the objective of sect A radicals. Hence, determining what the optimal (*p*_A_,*k*_A_) strategy is, is not necessarily straightforward.

For the symmetric case we are considering in this sub-section, the objective function for both sects reduces to

4.16 $$U(p,k)=(1-k)r^{\mathrm{eq}}.$$

Despite its simple form, much can be understood by maximizing equation (4.16). First, we note that, for a fixed value of *k*<1, maximizing *U*(*p*,*k*) over *p* is equivalent to maximizing *r*^eq^=*r*_1_ in equation (4.12) over *p*. However, *r*_1_ is an increasing function of *p* for any *k*<1, leading the optimal *p* to be chosen as large as possible, which is along the constraint line equation (4.8). We can thus recast our optimization problem in one dimension along the constraint line *k*=1−*p*/*ω*, using either *p* or *k* as an independent variable; we choose *p* for this task. Equation (4.16) can thus be rewritten as:

4.17 $$U(p)=\frac{p+1-\lambda }{2\omega }+\sqrt{\frac{p}{\omega^{2}}\left(\frac{1}{K(p)}-\frac{1}{1-p/\omega }\right)}.$$

At any point within the feasible sets shown in the shaded areas of figure 1*a*,*b*, *U*(*p*) is well defined. In order to determine whether a maximum exists, note that *U*′(*p*) will diverge at any endpoints of the feasible sets where the constraint line intersects the k=K(p) curve, due to the square root term. Additionally, if these endpoints are approached from below (increasing *p*) the derivative will diverge to −∞, while if approached from above (decreasing *p*) the derivative will diverge to +∞. For λ>1, the feasible set is 0≤*p*≤*p*_max_. Here, *U*′(*p*=0)=1 and $U^{\prime }(p\to p_{\max}^{-})=-\infty$. Since the derivative is continuous in between these endpoints, there will be at least one value 0<p*<*p*_max_ between them where *U*′(p*)=0 and *U*(p*) attains a maximum. In this case, the optimal strategy is given by the values (p*,1−p*/*ω*) on the constraint line. Similarly, for λ≤1 since the feasible set (if it exists) lies in a *p*_min_≤*p*≤*p*_max_ range, $U^{\prime }(p\to p_{\min}^{+})=+\infty$ and $U^{\prime }(p\to p_{\max}^{-})=-\infty$. Once more, due to the continuity of *U*′(*p*) between these endpoints, there will be at least one value *p*_min_<p*<*p*_max_ for which *U*′(p*) is zero and *U*(p*) is a maximum. The end result of this analysis is that, for the objective function proposed in equation (4.16), the optimal strategy for radicals is not to use the largest feasible *p* nor the largest feasible *k*, but rather intermediate values that use all the resources *ω* and where enough attacks and propaganda are employed.

The above arguments for the existence of an optimal strategy on the interior of the feasible set fail if the initial conditions do not allow the radical population to evolve towards *r*^eq^=*r*_1_; for example, if the optimal (*p*,*k*) values determined above fall in the red-shaded domains of the feasible sets, and the initial conditions are such that *r*(*t*=0)=*δ*<*r*_2_, then at steady state *r*^eq^=0. For λ>1, however, radicals can take the following ‘two step’ approach to maximize their objective function. First, they may select *k*>1/λ and a corresponding *p* such that this temporary strategy falls within the blue-shaded area of figure 1*a*. Since in this regime $r(t\to \infty )=r_{1}>r_{2}$, once the radical population reaches a larger than *r*_2_ threshold, the strategy can be switched to the optimal one. From figure 1*a*, it is clear that this temporary strategy is characterized by a higher *k* and lower *p* than the optimal one. Hence, for λ>1, a radical group that starts with few members and that wishes to optimize objective function equation (4.15) should utilize relatively high violence *k* and low indoctrination *p* rates; later, as the group approaches its equilibrium size, it should progress towards more intense indoctrination and less violence. For λ≤1, however, if the initial size of the radical group *δ* is smaller than the smallest *r*_2_ in the feasible set, no strategies for the emergence of radical populations at steady state exist.

### 4.3. Strategy asymmetric sects

We now consider partially symmetric sects with equal intrinsic parameters λ,*μ*,*ω*, equal total population *N*_*i*_, and equal initial conditions *r*_A_(*t*=0)=*r*_B_(*t*=0)=*δ*. Radical factions, however, are now allowed to choose their (*p*_*i*_,*k*_*i*_), *i* =A,B strategies independently of each other, and possibly asymmetrically. We further assume that the chosen initial conditions lead to a non-zero *r*^eq^ at steady state for both groups, if the overall parameter set allows for a *r*^eq^≠0 to emerge. Finally, we assume that the utility function of each sect is still given by equation (4.15). The major motivation behind studying this particular case is to place our model within a game-theoretic scenario, whereby players (radical factions) choose their strategies independently of each other. By doing so, we can attempt to identify the Nash equilibria (NE) of the ‘game’, defined as combinations of strategies (*p*_A_,*k*_A_) and (*p*_B_,*k*_B_) whereby neither sect can increase its utility by unilaterally changing to a different strategy. That is, if $\left(p_{A}^{\mathrm{NE}},k_{A}^{\mathrm{NE}}\right)$ and $\left(p_{B}^{\mathrm{NE}},k_{B}^{\mathrm{NE}}\right)$ together represent a Nash equilbrium, then for all other possible strategies (*p*_A_,*k*_A_), $U_{A}(p_{A},k_{A};p_{B}^{\mathrm{NE}},k_{B}^{\mathrm{NE}})\leq U_{A}(p_{A}^{\mathrm{NE}},k_{A}^{\mathrm{NE}};p_{B}^{\mathrm{NE}},k_{B}^{\mathrm{NE}})$, and the same is true for sect B’s utility.

To determine the Nash equilibria of this game, we first note that equilibrium radicalization levels are given by the intersections of the two nullclines defined by equations (4.6) and (4.7)

4.18 $$\begin{array}{rl} r_{B} & =\frac{r_{A}}{k_{B}[\lambda +r_{A}(p_{A}+1-\lambda )-p_{A}r_{A}^{2}]}\\ \;\mathrm{and}\;r_{A} & =\frac{r_{B}}{k_{A}[\lambda +r_{B}(p_{B}+1-\lambda )-p_{B}r_{B}^{2}]}, \end{array}\}$$

where we have kept the distinction (*p*_*i*_,*k*_*i*_) between *i*=A,B sects.

We begin by considering the possibility of symmetric Nash equilibria whereby the two sects play the same strategy (*p*_*i*_,*k*_*i*_)=(*p*,*k*) for *i*=A,B, leading to the non-zero equilibrium radical population *r*_A_=*r*_B_=*r*^eq^ as given by equation (4.12). A necessary, but not sufficient, condition for any Nash equilibria is that the strategy chosen by each sect leads to a local maximum in its utility function, such that an infinitesimal unilateral strategy change would not lead to utility increase. Without loss of generality, we focus on sect A. To determine the effects of unilateral changes in its strategy on *U*_A_(*p*_A_,*k*_A_;*p*_B_,*k*_B_) we differentiate equation (4.15) with respect to *p*_A_ and *k*_A_ using equation (4.18) where *p*_B_ and *k*_B_ are kept constant. Once the derivatives are taken we calculate them at *p*_A_=*p*_B_=*p* and *k*_A_=*k*_B_=*k*. We find

4.19 $$\begin{array}{rl} \frac{\partial U_{A}}{\partial p_{A}} & =\frac{kr_{1}^{2}(1-r_{1})(1-k+\beta )}{\beta (\beta +2)}\\ \;\mathrm{and}\;\frac{\partial U_{A}}{\partial k_{A}} & =\frac{r_{1}(1-k-\beta k)}{k\beta (\beta +2)}, \end{array}\}$$

where *β* is defined as

4.20 $$\beta =pkr_{1}\sqrt{B^{2}-4C},$$

with *B*,*C* given in equation (4.10), and *r*_1_ in equation (3.6). Note that *β*≥0 and that since *r*_1_≤1 and *k*≤1, ∂*U*_A_/∂*p*_A_≥0; hence, any symmetric Nash equilibria must have maximal *p*, if all other parameters are fixed, and lie along the constraint line *k*_A_=1−*p*_A_/*ω* in equation (4.8). Utilizing the constraint we can evaluate the total derivative of *U*_A_ with respect to *p*_A_ as

4.21 $$\frac{dU_{A}}{dp_{A}}=\frac{\partial U_{A}}{\partial p_{A}}+\frac{\partial U_{A}}{\partial k_{A}}\frac{dk_{A}}{dp_{A}}$$

and, once calculated, express the right-hand side above as a function of *p*_A_=*p* and *k*_A_=1−*p*/*ω*. Using equation (4.19) we find

4.22 $$\frac{dU_{A}}{dp_{A}}=\frac{1}{2+\beta }\frac{dU(p)}{dp}+\frac{h(p)}{2+\beta }.$$

Here d*U*(*p*)/d*p* is the derivative of equation (4.17), and *h*(*p*) is defined as:

4.23 $$h(p)\equiv (1-p/\omega )r_{1}^{2}(p)[1-r_{1}(p)].$$

Note that in equation (4.23), *r*_1_(*p*) is written only in terms of *p* due to the constraint in equation (4.8), and that 0≤*h*(*p*)≤1. To find symmetric Nash equilbria in the current context, where we allow radical factions to independently choose their strategies (*p*_*i*_,1−*p*_*i*_/*ω*) for *i*=A,B and only later impose symmetry, we set d*U*_A_(*p*_A_)/d*p*_A_=0; in the symmetry-enforced case discussed in §4.2, where strategies are forced to be symmetric throughout the optimization phase, the condition for optimality was to set d*U*(*p*)/d*p*=0. The two are related via equation (4.22).

Since d*U*(*p*)/d*p*=0 at the symmetry-enforced optimum value 0<p*<*p*_max_ discussed earlier, and since *h*(p*)>0, necessarily d*U*_A_/d*p*_A_>0 at (p*,1−p*/*ω*). We can thus conclude that the optimal strategy in the symmetry-enforced case, where both radical factions choose strategy (p*,1−p*/*ω*), cannot also be a symmetric Nash equilibrium in the case where the two sects are free to choose their own strategy. In fact, following the above analysis, we have shown that if the two radical groups were playing the symmetry-enforced optimum at p*, each group would be tempted to *increase* its *p*, and therefore d*ecrease* its *k* due to the constraint in equation (4.8). Thus our game theoretic analysis shows that sects that carry symmetric intrinsic parameters, but that are free to choose indoctrination and attack strategies independently of each other, will be less violent than if they were colluding to enforce symmetric strategic choices.

We may still ask if there exists the possibility of a symmetric Nash equilibrium at p*<*p*_NE_<*p*_max_, for which d*U*_A_/d*p*_A_ must vanish so that *U*′(*p*_NE_)=−*h*(*p*_NE_). As discussed in the previous section *U*′(*p*) decreases continuously from 0 to −∞ for p*<*p*<*p*_max_, while −*h*(*p*) is continuous and bound between −1 and 0. Therefore, *U*′(*p*) and −*h*(*p*) must cross at least once for p*<*p*_NE_<*p*_max_, thus yielding at least one potential symmetric Nash equilibrium at *p*_NE_. Multiple, symmetric Nash equilibria could arise if the curves intersected more than once. While the argument above proves that there must exist one or more symmetric strategies that are at least potentially Nash equilibria, it does not conclusively show that they are so, as (*p*^NE^,1−*p*^NE^/*ω*) would have to be global maxima of *U*_A_(*p*_A_,1−*p*_A_/*ω*;*p*^NE^,1−*p*^NE^/*ω*), as a function of *p*_A_ with all other parameters fixed, and not merely local maxima. It also does not address the potential for Nash equilibria that are asymmetric in strategies, where $\left(p_{A}^{\mathrm{NE}},k_{A}^{\mathrm{NE}})\neq (p_{B}^{\mathrm{NE}},k_{B}^{\mathrm{NE}}\right)$. To explore these factors, we turn to numerical investigations. Here, we make the assumption that any Nash equilibria of the game will occur along the constraint curves of the two sects, as is true for symmetric Nash equilibria. We further assume that initial conditions always allow for a non-zero equilibrium radicalization value, if parameters allow for it. We proceed by determining for each radical faction the best response curve to the other’s strategy, and find the intersections of these best response curves, which are Nash equilibria. Note that this method provides only pure Nash equilibria, in which each sect plays a definite strategy, and does not include mixed strategy Nash equilibria that would allow radical factions to play probabilistic combinations of strategies.

**Figure 2.**
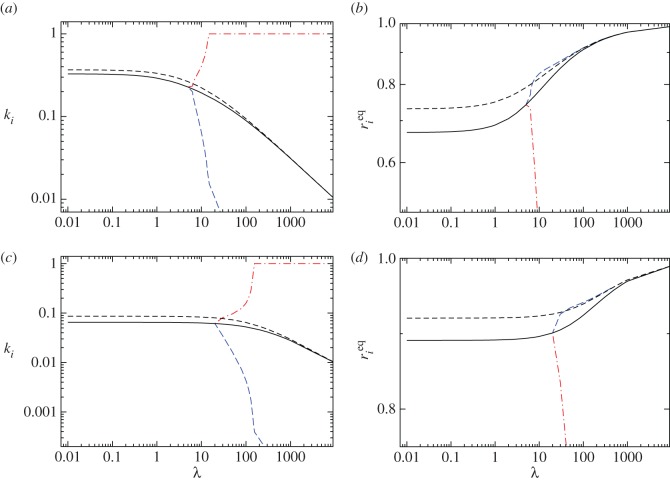
Numerically determined Nash equilibria (NE) for *i*=A,B asymmetric sects whose defining parameters and initial conditions are the same. All (*p*_*i*_,*k*_*i*_) strategies satisfy the constraint defined by equation (4.8) and may be chosen independently by the two sects. Dashed black curves represent the symmetry-enforced optimum strategy discussed in §4.2; solid black curves correspond to the symmetric Nash equilibria; long-dashed blue and dash-dot red curves correspond to the asymmetric Nash equillibria for the two sects. Note that the latter asymmetric Nash equillibria emerge only for large enough values of λ. (*a*,*c*) The Nash equilibria attack rate *k*^*NE*^_*i*_ for *i*=A,B; the corresponding indoctrination rates may be derived from the constraint *p*_*i*_=*ω*(1−*k*_*i*_). (*b*,d**) The relative equilibrium radical population $r_{i}^{\mathrm{eq}}$ for *i*=A,B. In (*a*,*b*) *ω*=16 and in (*c*,d**) *ω*=160.

We present our results in figure 2 where the Nash equilibria values for *k*_*i*_ and the corresponding values of $r_{i}^{\mathrm{eq}}$ are determined as a function of λ for *i*=A,B. These results, obtained for symmetric sects free to play their own strategies (*p*_*i*_,*k*_*i*_=1−*p*_*i*_/*ω*), are compared and contrasted with those obtained in §4.2 where the (p*,1−p*/*ω*) strategies were constrained to be symmetric. We examine the case of small and large overall resource rates *ω*=16 and *ω*=160, respectively. As can be seen from all panels of figure 2, and as predicted, we do find symmetric Nash equilibrium that exhibit higher indoctrination rates *p*_NE_, lower attack rates *k*_NE_=1−*p*_NE_/*ω* and lower radical population ratios *r*_1_(*p*_NE_) compared with those evaluated at the symmetric optimum p*. In figure 2*a*,*c*, we also find that *k*_NE_ decreases from a finite value as λ→0 towards zero as λ→∞. At the same time, figure 2*b*,*c* reveal that the symmetric Nash equilibrium *r*_1_(*p*_NE_) is an increasing function of λ. Also note that for lower λ values, only the symmetric Nash equilibrium (*p*_NE_,*k*_NE_) exists, while higher λ values display an additional single asymmetric Nash equilibrium in which one sect is extremely violent, with high *k* values, and the other’s attack rate approaches zero. Owing to symmetry, which sect plays which role is arbitrary. Note, though, that the very violent sect is also characterized by a very small radical population, while the moderately violent sect is marked by a larger radical population, whose numbers may even exceed those found in the symmetric optimal case p*. We thus find that for large values of spontaneous radicalization λ, a radical group may achieve a Nash equilibrium with their rival group either through a small radical faction engaging in very violent activities, or through a large number of radicals performing only moderately violent aggressive acts towards the other sect as long as the other sect adopts the alternate role. Finally, we find that higher resource rates *ω* lead to lower levels of violence but higher radicalization at the Nash equilibra, as can be seen by comparing figure 2*c*,d** versus 2*a*,*b*.

### 4.4. Fully asymmetric sects

We now study the dynamics of equations (4.6) and (4.7) for arbitrary parameters. While a full analytical treatment is infeasible, we can find all equilibrium points by determining the intersections of the nullclines

$$\begin{array}{rl} r_{B} & =\frac{r_{A}}{xk_{B}[\lambda_{A}+r_{A}(p_{A}+x-\lambda_{A})-p_{A}r_{A}^{2}]}\\ r_{A} & =\frac{x\mu_{B}r_{B}}{k_{A}[\lambda_{B}+r_{B}(p_{B}+\mu_{B}x-\lambda_{B})-p_{B}r_{B}^{2}]}. \end{array}$$

Upon substitution and after discarding the trivial solution *r*_A_=*r*_B_=0, we find the following quartic equation for *r*_A_

4.24 $$c_{4}r_{A}^{4}+c_{3}r_{A}^{3}+c_{2}r_{A}^{2}+c_{1}r_{A}+c_{0}=0$$

where

4.25 $$\begin{array}{rl} c_{4} & =\lambda_{B}p_{A}^{2},\\ c_{3} & =-2\lambda_{B}p_{A}(p_{A}+x-\lambda_{A})-\frac{p_{A}}{xk_{B}}(p_{B}+\mu_{B}x-\lambda_{B}),\\ c_{2} & =-2\lambda_{B}\lambda_{A}p_{A}+\lambda_{B}{\left(p_{A}+x-\lambda_{A}\right)}^{2}\\ & \;+\frac{1}{xk_{B}}(p_{B}+\mu_{B}x-\lambda_{B})(p_{A}+x-\lambda_{A})-\frac{p_{B}}{x^{2}k_{B}^{2}}+\frac{p_{A}\mu_{B}}{k_{A}k_{B}},\\ c_{1} & =2\lambda_{B}\lambda_{A}(p_{A}+x-\lambda_{A})+\frac{\lambda_{A}}{xk_{B}}(p_{B}+\mu_{B}x-\lambda_{B})\\ & \;-\frac{\mu_{B}}{k_{A}k_{B}}(p_{A}+x-\lambda_{A}),\\ c_{0} & =\lambda_{B}\lambda_{A}^{2}-\frac{\lambda_{A}\mu_{B}}{k_{A}k_{B}}. \end{array}\}$$

Equation (4.24) has at most four non-trivial solutions that can be found explicitly using the quartic formula. Not all of them are physically acceptable since we must impose 0≤*r*_*i*_≤1 for *i*=A,B. By constructing a trapping region and applying the Poincaré–Bendixson Theorem [27], we can, however, find parameters that will guarantee the existence of at least one non-trivial, physically acceptable, equilibrium point for the two competing sects. The boundary of [0,1]×[0,1] in (*r*_A_,*r*_B_) space almost works as a trapping region, with the exception of the corner at the origin. To construct our trapping region, we must then show that the [0,1]×[0,1] box from which the origin is excised will still trap trajectories. In particular, we must show that the vector field along the boundary of our trapping region points into the trapping region itself so that any trajectory that starts within our trapping region will never leave it. It is simple to show that if *r*_A_=1 then ${\dot{r}}_{A}<0$ and if *r*_A_=0,*r*_B_≠0 then ${\dot{r}}_{A}>0$. If *r*_A_=*r*_B_=1, then both ${\dot{r}}_{A}<0$ and ${\dot{r}}_{B}<0$ hold. Similarly, if *r*_A_=0 and *r*_B_=1, then ${\dot{r}}_{A}>0$ and ${\dot{r}}_{B}<0$. Similar relationships hold upon exchanging the A,B labels. We now show that for certain parameter values, the equilibrium at the origin can be made unstable. We linearize equations (4.6) and (4.7) near the origin to determine its stability. The Jacobian matrix *J* at (0,0) is given by

4.26 $$J=\left[\begin{array}{cc} -1 & x\lambda_{A}k_{B}\\ \lambda_{B}k_{A}/x & -\mu_{B} \end{array}\right]$$

and has at least one stable eigendirection, since its trace is negative. The origin will be an unstable saddle point if the determinant, the product of the eigenvalues, is negative, a condition that is satisfied for λ_A_λ_B_*k*_A_*k*_B_>*μ*_B_. A simple matrix calculation shows that the stable eigendirection lies in the second and fourth quadrant, out of our trapping region. Hence, the condition λ_A_λ_B_*k*_A_*k*_B_>*μ*_B_ is enough to guarantee that trajectories close to the origin in the first quadrant are repelled away from it. Since they are also trapped in the (0,1]×(0,1] region, at least one physically relevant equilibrium must exist. The constraint λ_A_λ_B_*k*_A_*k*_B_>*μ*_B_ is the asymmetric analogue to the condition *k*>1/λ from §4.1. Specifically, if both attack and spontaneous radicalization rates are high enough, a non-zero radical population will persist for all time, regardless of initial condition.

Though completely understanding all facets of the problem for general asymmetric parameters would be impractical, we can focus on a few key parameters that might shed light on some of the fundamental behaviours of the system in the general case. In particular, we consider sects that have different levels of ‘strength’, measured by *N* (strength in numbers) and *ω* (strength in resources). Hence, we will compare and contrast sects with large and small *N*_*i*_, and large and small *ω*_*i*_, for *i* = A,B while keeping the spontaneous radicalization and de-radicalization rates the same, so that λ_A_=λ_B_=λ and *μ*_B_=1 in the dimensionless equations (4.6) and (4.7). We begin by numerically determining the pure Nash equilibria of the system using the same approach as described above. We select specific parameter values that allowed for both symmetric and asymmetric Nash equilibria to arise in §4.3: *ω*=16 and λ=10.

**Figure 3.**
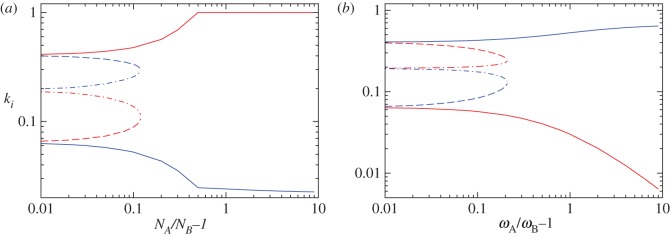
Numerically determined Nash equilibria (NE) for asymmetric sects whose defining parameters and initial conditions are the same with the exception of either their sizes *N*_A_>*N*_B_ (*a*) or their resources *ω*_A_>*ω*_B_ (*b*). All (*p*_*i*_,*k*_*i*_) strategies satisfy the constraint defined by equation (4.8). The blue curves correspond to the (numerically or resource-wise) weaker sect B and the red curves to the stronger sect A. The solid, dashed and dash-dotted lines indicate three distinct (*k*_A_,*k*_B_) equilibria pairs; for instance the solid red and solid blue curves together describe the most asymmetric outcome. Parameters are set at λ=10, and *ω*_A_=*ω*_B_=*ω*=16 in (*a*); and at λ=10, *ω*_B_=16 in (*b*). Note that since *N*_A_ and *N*_B_ always appear as a ratio in equation (4.15) and in the definition of *x*, we do not need to explicitly set their numerical values in (*b*) where *N*_A_=*N*_B_.

First, we explore how the Nash equilibria of the system change when sects have the same resources *ω*_A_=*ω*_B_=*ω* but different sizes *N*_A_>*N*_B_, so that *x*<1 in equation (4.4). Our results are shown in figure 3*a* where we plot the Nash equilibria attack rates *k*_*i*_, for *i*=A,B corresponding to a given size asymmetry *N*_A_/*N*_B_−1>0. The corresponding values of *p*_*i*_ can be obtained through the constraint line in equation (4.8). As can be seen from figure 3*a* if the size asymmetry is small, three separate Nash equilibria exist: one near the symmetric Nash equilibria (dash-dotted lines), and two near the possible asymmetric Nash equilibria (dashed and solid lines) discussed previously. For asymmetries approaching 10% in size, however, the Nash equilibria that corresponded to the symmetric case approaches one of the asymmetric Nash equilibria and they annihilate, leaving only a single pure Nash equilibria in which the radical faction of the larger sect A is more engaged in violent attacks than radicals from the smaller sect B. Upon evaluating the corresponding radical populations *r*_*i*_ for *i*=A,B at the Nash equilibria we find that *r*_A_≪*r*_B_. These results are a direct consequence of the large size asymmetry and the definition of the utility functions *U*_*i*_, for *i* = A,B. The larger sect A will be mostly concerned with maximizing *r*_A_, while the focus of sect B will be to minimize the violence from sect A radicals. Since the only way for sect B to reduce attacks from A is to decrease *r*_A_ by minimizing its own attacks, sect B’s optimal strategy is to choose low *k*_B_ values, as our numerical Nash equilibria estimates show. On the other hand, *r*_A_ will increase with increasing *r*_B_, which can be spurred by sect A radicals employing a large attack rate *k*_A_. Our current analysis leads to the conclusion that larger sects are expected to carry a smaller population of radicals who are very violent, while smaller sects cannot afford extreme levels of violence, as they would be the target of repeated attacks inflicted by their opponent.

In figure 3*b*, we explore how the Nash equilibria of the system change when the sects have the same sizes *N*_A_=*N*_B_=*N* but different resource availability *ω*_A_>*ω*_B_ so that in equation (4.4), *x*<1. Results are similar to those described in figure 3*a*, with the roles of the two sects switched. Large *ω*_A_/*ω*_B_−1 asymmetries lead radicals from the weaker group B to display greater violence than those from the more resourceful sect A; similarly the weaker group is less radicalized than the stronger one. The resource asymmetry does not directly affect the utility functions the same way the size asymmetry did, but does play a role through the resource constraint (4.8). A relatively large *ω*_A_ allows for larger indoctrination *p*_A_, which will lead to an increase in *r*_A_, as desired in the utility function, and to a decrease in *k*_A_ due to equation (4.8).

### 4.5. Optimal control for competing sects

We have thus far ignored a critical aspect of the formation and stabilization of radical factions. Specifically, our model does not include a sect’s ability to organize or to optimize a strategy in response to an evolving situation and then share this strategy with its members. However, the ability to quickly disseminate strategic decisions to a group’s constituency is essential for radical extremists to establish power. Many revolutions arose from student groups where a large body of similarly idealistic and disillusioned people could rapidly circulate ideas through physical or virtual proximity, such as in the case of the 1979 Iran Revolution, or the formation of the Taliban in Afghanistan in the late 1970s [28]. As a more modern example, the Islamic State in Syria and Iraq has used social media to effectively spread their radical agenda [22]. Different groups may greatly vary in their ability to organize attacks and disseminate propaganda, even as the parameters used in our model to quantify them may be the same. We now introduce a small variant in equations (4.6) and (4.7) to include a faction’s ability to organize and disseminate policy in the dynamic framework for radical faction formation and consolidation.

We start by assuming that sects A and B have different power structures and organizational capabilities. To be concrete, we assume that at time *t*<*t*_0_ radical factions of the two sects begin with random, possibly asymmetric strategies. At time *t*=*t*_0_ sect B radicals unilaterally modify their strategy to optimize their utility given the current strategy used by sect A radicals; for example, at *t*=*t*_0_, sect B radicals may increase their attack rate and decrease indoctrination. Sect A radicals will notice this change, craft an optimal response strategy, coordinate and mobilize their members, and finally enact their new, optimal policy. We denote the time interval between sect B’s strategy change and sect A’s response by Δ_A_ so that sect A’s new strategy is in effect at *t*_1_=*t*_0_+Δ_A_. Sect B radicals may now modify their strategy in return, optimizing their attack and propaganda parameters in response to the new policies enacted by sect A. We assume changes in sect B’s strategies are enacted at time *t*_2_=*t*_1_+Δ_B_, where Δ_B_ is the equivalent time for sect B to reorganize after sect A’s strategic changes. Since decision and implementation times for the two sects may differ we allow Δ_A_≠Δ_B_. Sect dynamics thus unfold according to equations (4.6) and (4.7) under initial values $\left(p_{A}^{0},k_{A}^{0}\right)$ selected randomly and $\left(p_{B}^{0},k_{B}^{0}\right)$ determined as the optimal strategy in response to $\left(p_{A}^{0},k_{A}^{0}\right)$ at time *t*=*t*_0_. The system continues to evolve via a cascade of alternating dynamic strategic updating at *t*_*j*_=*t*_0_+[(*j*+1)/2]Δ_A_+[*j*/2]Δ*B*, where [.] is the integer part. We can thus define a sequence of epochs *τ*_*j*_=*t*_*j*+1_−*t*_*j*_ during which strategies $\left(p_{i}^{j},k_{i}^{j}\right)$ for *i* = A,B are used.

The framework we have just introduced is related to two well-established game theoretic scenarios. The first is that of Stackleberg games, where a preset leader and follower are defined: the leader’s strategy is optimized with respect to how the follower is expected to respond; the follower optimizes its strategy in response to the leader’s [29]. The second scenario is that of mean field games [30–33]: partial differential equations used to understand the behaviour of many interacting agents, where each is seeking to optimize a given function but is also constrained by the choices of others. In general, mean field games may involve solving both a forward-in-time propagation problem and a backwards-in-time optimization one.

Our optimization problem is somewhat of a hybrid between a Stackleberg and a mean field game, with leaders and followers alternating and behaviours described via the differential equations in equations (4.6) and (4.7). To complete its formulation, we must introduce the proper objective function to be maximized. Before doing so, we note that since the *τ*_*j*_ epochs are finite and the strategies within it are enacted for a finite time period, an equilibrium state will not be reached within any *τ*_*j*_ time interval. Since sect B radicals are the first to optimize their strategy, we introduce the following objective function for sect B over the *τ*_*j*_ epoch

4.27 $$U_{B}^{j}(p_{B},k_{B})=\int_{t_{j}}^{t_{j+1}}\left[{\dot{r}}_{B}-\frac{N_{A}}{N_{B}}k_{A}{\dot{r}}_{A}\right]dt,$$

where *k*_B_ is subject to the constraint in equation (4.8), and where *j* is even. Specifically, we assume that sect B radicals have knowledge of sect A’s intrinsic parameters and strategic choices, including their reaction times Δ_A_. At time *t*_*j*_ sect B radicals modify their (*p*_B_,*k*_B_) strategies by maximizing the integral in equation (4.27) over the future time interval ending at *t*_*j*+1_=*t*_*j*_+Δ_A_, when sect A radicals are expected to react to such modifications. The values of *j* in equation (4.27) are even, since the first period of optimization begins at *t*_0_ and ends at *t*_1_=*t*_0_+Δ_A_, alternating with sect A in future cycles. A similar objective function $U_{A}^{j}$ can be defined for sect A by interchanging the A,B labels and using *j* odd as the first optimization interval for sect A radicals begins at *t*_1_ and ends at *t*_2_=*t*_1_+Δ_B_.

We first analyse two distinct limits. If $\Delta_{B}\to \infty$ the objective function defined in equation (4.27) will be dominated by near equilibrium dynamics and $U_{B}^{j}(p_{B},k_{B})$ can be approximated as

4.28 $$U_{B}^{j}\approx \left[r_{B}^{\mathrm{eq}}-\frac{N_{A}}{N_{B}}k_{A}r_{A}^{\mathrm{eq}}\right]-\left[r_{B}(t_{j})-\frac{N_{A}}{N_{B}}k_{A}r_{A}(t_{j})\right].$$

Optimizing equation (4.27) is thus equivalent to optimizing equation (4.15) as the quantities evaluated at the lower bound *t*_*j*_ are fixed and do not depend on the (*p*_B_,*k*_B_) values we are optimizing over. Similar considerations hold for $U_{A}^{j}$ if $\Delta_{A}\to \infty$. In the opposite limits Δ_A_≪1 and Δ_B_≪1 equation (4.27) can be approximated as

4.29 $$U_{B}^{j}\approx \Delta_{A}\left({\dot{r}}_{B}-\frac{N_{A}}{N_{B}}k_{A}{\dot{r}}_{A}\right).$$

Since Δ_A_ is fixed, our task is to maximize the term in parenthesis in equation (4.29) through equations (4.6) and (4.7) under the constraint defined in equation (4.8). The resulting equation is linear in *k*_B_ and *p*_B_, so it can be trivially shown that if *k*_A_,*r*_A_≠0 and *r*_B_≠0,1, equation (4.29) is maximized by (*k*_B_,*p*_B_)=(0,*ω*_B_) for all *j*. If *k*_A_=0, however, all strategy decisions for B are equivalent, and regardless of which strategy is chosen, *r*_B_ will exponentially decay to zero. Similarly, we can show that under the same conditions described above for sect B, sect A radicals will follow an analogous updating strategy (*k*_A_,*p*_A_)=(0,*ω*_A_). As a result, for short update times, we expect both sects to very quickly fix their strategies to display no violence. The corresponding propaganda values will set at arbitrary values. In this case, there is no dynamic strategy updating and eventually the two radical groups disappear. Note that here our game defines a classic Prisoner’s Dilemma: individually, each *i*=A,B sect maximizes its utility by selecting *k*_*i*_=0, but if both sects choose the no attack strategy, the outcome is less optimal for both than if they had chosen *k*_*i*_≠0.

**Figure 4.**
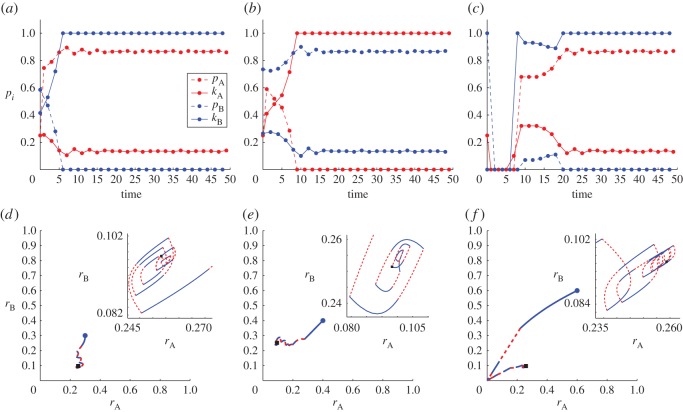
Time-dependent optimal strategies and radical population dynamics for asymmetric sects whose defining parameters and initial radical population sizes are the same. Initial conditions are set to $r_{i}^{0}=0.3$ in (*a*,d**), to $r_{i}^{0}=0.4$ in (*b*,*e*) and to $r_{i}^{0}=0.6$ in (*c*,*f*) for *i*= A,B. Non-dimensional parameters are fixed at Δ_*i*_=*μ*_B_=1, λ_*i*_=3, *N*_*i*_=1000, *ω*_*i*_=1 for *i*=A,B; initial strategic conditions are set as $k_{A}^{0}=0.25,\;p_{A}^{0}=0.25$ in all panels. (*a*–*c*) Optimal strategies $\left(p_{i}^{j},k_{i}^{j}\right)$ for *i*=A,B evolving over the *τ*_*j*_ time epochs. Circles represent strategic changes. The blue coloured B sect is the first to optimize its strategy at time *t*_0_. For all parameter choices displayed here, one sect tends to display more violence and less indoctrination than the other. (d**–*f*) Resulting $\left(r_{A}^{j},r_{B}^{j}\right)$ phase plane dynamics. Initial conditions are marked with a blue circle; endpoints with a black square. The solid blue line refers to a sect B update over a time period Δ_A_, the dashed red curve corresponds to a sect A update over a time period Δ_B_. The inset magnifies the phase diagram in the later stages of the optimization process as the system approaches the black square endpoint. In (*a*,d**) and (*b*,*e*), we used a finer grid for the optimization search (200 points) than in (*c*,*f*) (100 points). The size of the corresponding limit cycles in the three insets does not seem to be affected by the finer or coarser grid sizes used during the optimization.

**Figure 5.**
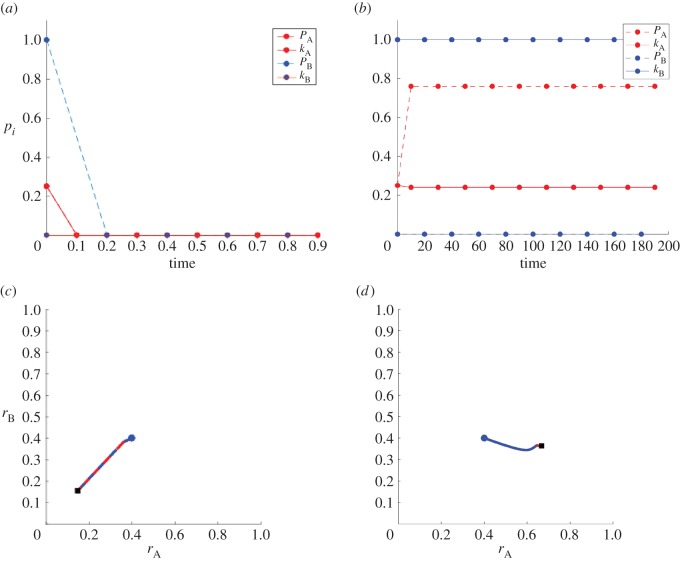
Time-dependent optimal strategies and radical population dynamics for asymmetric sects whose defining parameters and initial radical population sizes are the same. Organizational times are set at Δ_*i*_=0.1 in (*a*,*c*) and at Δ_*i*_=10 in (*b*,d**) for *i*=A,B. Initial conditions are chosen as $r_{i}^{0}=0.4$. Other non-dimensional parameters and initial strategic conditions are set as in figure 4. (*a*,*b*) optimal $\left(p_{i}^{j},k_{i}^{j}\right)$ strategies evolving over the *τ*_*j*_ time epochs. The notation is the same as in figure 4. (*c*,d**) Resulting $\left(r_{A}^{j},r_{B}^{j}\right)$ phase plane dynamics. Figure 4*b*,*e* may be used to compare and contrast the above plots with the intermediate organizational time Δ_*i*_=1.

**Figure 6.**
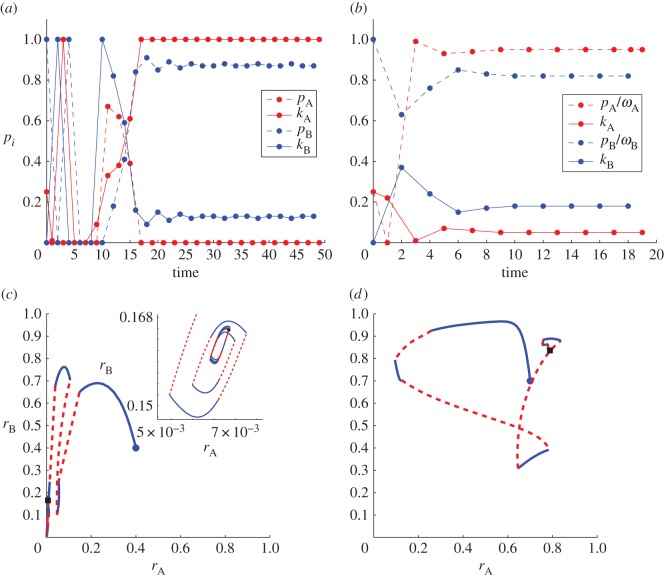
Time-dependent optimal strategies and radical population dynamics for sects whose initial radical population sizes and defining parameters are the same, with the exception of their sizes *N*_*i*_ (*a*,*c*) or their resource availability *ω*_*i*_ (*b*,d**). (*a*,*b*) optimal $\left(p_{i}^{j},k_{i}^{j}\right)$ strategies over several time epochs *τ*_*j*_; (*c*,d**) resulting $\left(r_{A}^{j},r_{B}^{j}\right)$ phase plane dynamics. In all panels, Δ_*i*_=*μ*_B_=1, λ_*i*_=3, and $\left(p_{A}^{0},k_{A}^{0})=(0.25\omega_{A},0.25\right)$. In (*a*,*c*), differential sect sizes are *N*_A_=1000 and *N*_B_=100; available resources are equal and set at *ω*_*i*_=1 for *i*=A,B. In (*b*,d**), sect sizes are the same at *N*_*i*_=1000 for *i*=A,B and differential resources are set at *ω*_A_=160 and *ω*_B_=32. The notation is the same as in figure 4. The inset magnifies the phase diagram in the later stages of the optimization process, as the system approaches the black square endpoint.

**Figure 7.**
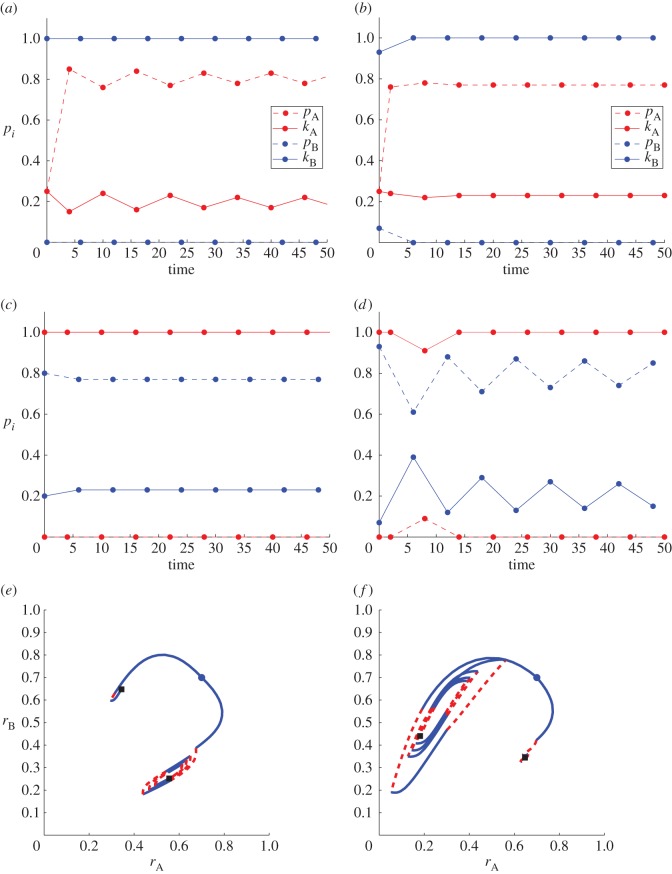
Time-dependent optimal strategies and radical population dynamics for asymmetric sects whose initial radical population sizes and defining parameters are the same, with the exception of their organizational times Δ_*i*_. In all panels, parameters are *μ*_B_=1, λ_*i*_=3, *N*_*i*_=1000, *ω*_*i*_=1 for *i*=A,B and initial conditions are set as $r_{A}^{0}=r_{B}^{0}=0.7$. (*a*–d**) Optimal $\left(p_{i}^{j},k_{i}^{j}\right)$ strategies over several time epochs *τ*_*j*_. In (*a*) $\left(p_{A}^{0},k_{A}^{0})=(0.25,0.25\right)$ and Δ_A_=4,Δ_B_=2; in (*b*) $\left(p_{A}^{0},k_{A}^{0})=(0.25,0.25\right)$ and Δ_A_=2,Δ_B_=4; in (*c*) $\left(p_{A}^{0},k_{A}^{0})=(0,1\right)$ and Δ_A_=4,Δ_B_=2; in (d**) $\left(p_{A}^{0},k_{A}^{0})=(0,1\right)$ and Δ_A_=2,Δ_B_=4. (*e*,*f*) Each panel traces two distinct trajectories corresponding to different initial strategies for sect A. Panel (*e*) contains the two $\left(r_{A}^{j},r_{B}^{j}\right)$ trajectories corresponding to the $\left(p_{i}^{j},k_{i}^{j}\right)$ optimal strategies shown in panels (*a*) and (*c*), both characterized by Δ_A_=4, Δ_B_=2. The trajectory that veers from the initial point towards the right corresponds to $\left(k_{A}^{0},p_{A}^{0})=(0.25,0.25\right)$ and to (*a*); the one that veers towards the left to $\left(k_{A}^{0},p_{A}^{0})=(0,1\right)$ and to (*c*). Similarly, the trajectories in (*f*) are characterized by Δ_A_=2, Δ_B_=4. Here, the trajectory that veers from the initial point towards the right corresponds to $\left(k_{A}^{0},p_{A}^{0})=(0.25,0.25\right)$ and to (*b*); the one that veers towards the left to $\left(k_{A}^{0},p_{A}^{0})=(0,1\right)$ and to (d**).

Intermediate values of Δ_*i*_ for *i*=A,B yield intricate dynamics as can be seen in figures 4–7. In these figures, all optimizations were performed using a grid search with typically 100 points along the (*p*_*i*_,*k*_*i*_) axes for *i*=A,B, except where otherwise indicated. Thus, at each epoch *τ*_*j*_, radical factions may select their new strategy from a space of 100×100 (*p*_*i*_,*k*_*i*_) possible configurations subject to the constraint in equation (4.8). We select *t*_0_=0 and assume that the initial strategy for sect A radicals is set at (*p*_0_,*k*_0_)=(0.25,0.25) unless otherwise specified.

In figure 4, we show the results of our alternating optimization process for Δ_*i*_=1 and for several sets of symmetric initial conditions $r_{i}^{0}=0.3,0.4,0.6$ for *i*=A,B. All other parameters are kept the same and are explicitly given in the figure caption. In figure 4*a–c*, we plot the optimal values of $\left(p_{i}^{j},k_{i}^{j}\right)$ over each *τ*_*j*_ epoch. As can be seen, one sect will select higher indoctrination, the other higher violence depending on the initial conditions and in ways that are not easily predictable. The two sects selecting opposite strategic choices is a general trend that persists for other initial conditions and parameters not shown here, similar to the asymmetric Nash equilibria discussed in §4.4. It is interesting to note that while we do not impose that the (*p*_*i*_,*k*_*i*_) strategies must lie on the constraint line defined by equation (4.8), the radical factions will typically maximize their utility functions by settling on the constraint line. Indeed, apart from a few initial time epochs *τ*_*j*_, the constraint $k_{i}^{j}+p_{i}^{j}/\omega_{i}=1$ is satisfied throughout the optimization process for all *j* and for *i*=A,B. In figure 4d*–f*, we follow the dynamics of the radical populations and note that the system will evolve towards final values that depend dramatically on initial conditions. In particular, by comparing figure 4*a*–*c* and 4d**–*f*, we note that sects with a larger radical faction are associated with lower levels of violence and vice versa. We find that this too is a general, robust trend that persists over initial conditions and parameters choices, again falling in line with what was observed in §4.4. Finally, one common feature of the long time dynamics observed in all panels of the lower row of figure 4 is a push–pull trend between the two sects that leads to limit cycles, as can be seen in the insets. The exact size of these limit cycles may depend on our coarse optimization routine. To examine this possibility we allowed different grid sizes while searching for the optimal strategies. In particular, the grid size used in figure 4*a*,d** and 4*b*,*c* is twice as large (200 points) as that used in 4*c*,*f* (100 points). We notice that the typical width of the limit cycles in all three panels are comparable, indicating that the grid discretization plays a minor role in the emergence of the limit cycles shown in figure 4.

To study how the symmetric organizational time scales Δ_*i*_ affect sect dynamics we plot the optimal $\left(k_{i}^{j},p_{i}^{j}\right)$ for various Δ_*i*_ values for *i*=A,B in figure 5. In particular, in figure 5*a*,*c*, we set Δ_*i*_=0.1 while in figure 5*b*,d** Δ_*i*_=10. Initial conditions are fixed at $r_{i}^{0}=0.4$ for *i*=A,B. The resulting curves should be compared with the ones displayed in the central panels of figure 4 for which Δ_*i*_=1 with all other parameters and initial conditions the same as in figure 5. Figure 5*b*,d** confirm that strategic choices are dominated by near equilibrium behaviour for very large Δ_*i*_ values, while figure 5*a*,*c* show that very small Δ_*i*_ yield *k*_*i*_≈0 for both groups, as anticipated above. Note that although in figure 5*a*, we stop at *t*=1 and the corresponding endpoint in figure 5*c* is (*r*_A_,*r*_B_)=(0.15,0.19) upon letting t→∞ asymptotically *r*_*i*_→0 for *i*=A,B.

In figure 6, we explore how asymmetries in size *N*_*i*_ or resources *ω*_*i*_ between the *i*=A,B sects affect the optimization dynamics, similar to our exploration of these effects with regard to Nash equilibria. In figure 6*a*,*c*, we set *N*_B_<*N*_A_ and *ω*_A_=*ω*_B_, while in figure 6*b*,d**, we fix *ω*_B_<*ω*_A_ and *N*_A_=*N*_B_. As in the Nash equilibrium case, the more populous sect displays greater levels of violence but a smaller percentage of radicalized members, while the opposite is true for the sect with greater resources.

The effects of radical factions with different organizational times are explored in figure 7 where Δ_A_≠Δ_B_, and where different values for $\left(p_{A}^{0},k_{A}^{0}\right)$ are selected. We set *ω*_*i*_=1 for *i*=A,B and do not require initial strategies to fall on the constraint line defined by equation (4.8). In figure 7*a*,*c*,*e*, Δ_A_=4, Δ_B_=2, while in figure 7*b*,d**,*f*, Δ_A_=2,Δ_B_=4. For each of these choices, we use two sets of initial strategies $\left(p_{A}^{0},k_{A}^{0})=(0.25,0.25\right)$ in figure 7*a*,*b*, or (0,1) in figure 7*c*,d**. These choices yield four sets of optimal $\left(p_{i}^{j},k_{i}^{j}\right)$ trajectories for *i*=A,B over various *τ*_*j*_ epochs. We find that the initial strategy chosen by sect A radicals highly affects the dynamics. For $\left(p_{A}^{0},k_{A}^{0})=(0.25,0.25\right)$, sect B radicals select highly aggressive strategies with $k_{B}^{j}≃1$ and $p_{B}^{j}≃0$ for large enough *j*, regardless of the reorganizational times Δ_*i*_ for *i* = A,B. Sect A radicals on the other hand select high indoctrination rates $p_{A}^{j}$, while their $k_{A}^{j}$ attack rates hover around the initial value $k_{A}^{0}=0.25$. Vice versa, initial strategies $\left(p_{A}^{0},k_{A}^{0})=(0,1\right)$ lead to sect B radicals opting for a mixture of indoctrination and attack rates with $\left(p_{B}^{j},k_{B}^{j})≃(0.8,0.2\right)$, while sect A’s strategy remains one of extreme violence. Once more, this is true regardless of Δ_*i*_ for *i*=A,B. The time delay asymmetries, however, do affect the oscillatory nature of the optimization process, as can be seen from comparing figure 7*a*,*b* and especially figure 7*c*,d**.

In figure 7*e*, we follow the $\left(r_{A}^{j},r_{B}^{j}\right)$ trajectories corresponding to figure 7*a*,*c* over several time epochs *τ*_*j*_. Similarly, figure 7*f* shows the $\left(r_{A}^{j},r_{B}^{j}\right)$ dynamics corresponding to figure 7*b*,d**. Initial radical population sizes are set at $\left(r_{A}^{0},r_{B}^{0})=(0.7,0.7\right)$ and are denoted by a blue circle in all cases. Black squares indicate the ending points of the various trajectories. In figure 7*e*, the path that veers towards the right and proceeds to define a limit cycle is characterized by $\left(p_{A}^{0},k_{A}^{0})=(0.25,0.25\right)$, corresponding to figure 7*a*; the one that veers towards the left is associated with $\left(p_{A}^{0},k_{A}^{0})=(0,1\right)$ and corresponds to figure 7*c*. As seen in the Nash equilibrium case, the sect choosing more violence is less radicalized than the sect choosing more propaganda, in both cases. We find similar behaviour in figure 7*f*. Here the trajectory that veers to the right corresponds to $\left(p_{A}^{0},k_{A}^{0})=(0.25,0.25\right)$ and to figure 7*b*; the one that veers towards the left is associated with $\left(p_{A}^{0},k_{A}^{0})=(0,1\right)$ and to figure 7d**.

Although we have only shown a few characteristic cases, solutions arising from the alternating optimization process typically converge to a Nash equilibrium or to a limit cycle around a Nash equilibrium; the qualitative features of these solutions generally match the behaviour found from identifying the Nash equilibria as described in §4.4. We can conclude that whether a repeated and alternating, or a simultaneous Nash equilibrium process is used to determine optimal behaviour, sects with smaller radical fractions will typically attack their opponents at higher rates, while sects with larger radical factions prefer a split strategy characterized by stronger indoctrination.

## 5. Conclusion

In this work, we studied the radicalization of two rival sects, each characterized by moderate and extreme factions. We included spontaneous radicalization and de-radicalization, as well as active indoctrination and the perpetration of violent attacks towards the other sect. Actions taken by each group are modulated by a sensitivity factor that may or may not depend on the actions taken by the opposite one. Using a set of coupled ODEs, we analysed non-interacting and interacting but symmetric sects, outlined behavioural trends and, where possible, derived analytical solutions. A game theoretical approach was then introduced, whereby all parameters are fixed except for internal indoctrination and external attack rates. These are the ‘strategies’ that radical factions of each sect adjust to optimize a given utility function meant to increase their own ranks and decrease rival attacks. Indoctrination and attack rates are also subject to a constraint limiting the total available resources available so that one activity may be favoured only at the expense of the other. We analysed the game in terms of Nash equilibria as well as through a simple iterative process that included possibly different response times.

One of our main findings is that, unless very high rates of violence are employed, small groups of radicals in overall moderate sect populations cannot be sustained over long times. Eventually, radicals will become less extreme and the entire sect will consist of moderates. Our results may thus offer some perspective on the mechanisms that lead radical groups who tend to employ greater violence in their early days, when they are still numerically small, to transition towards less violent methods, such as indoctrination, later on, as they mature. We also find that sects whose non-strategic parameters are completely symmetric allow for Nash equilibria that are not symmetric in their indoctrination and attack rate strategies. Of the two competing sects, our results show that the radical faction of one becomes less numerous but very violent, while the other sect is characterized by a large number of fanatics who are less violent.

This dichotomy between sect size and level of violence will persist if we relax the condition of symmetric non-strategic parameters and also allow the two sects to differ in total population size. In this case, the more populous sect will display a smaller percentage of very violent radicals than the less populous one. Why does this happen? If radicals from the less populous sect chose to use high levels of violence themselves, they would be subject to too many retaliatory attacks from their much larger opponent, so their best strategy is to favour internal propaganda over direct attacks of their rivals. On the other hand, radicals of the more populous sect are constantly seeking ways to increase their ranks among moderates of their own sect. For them, increasing the level of violence against the less numerous sect is a way to incense moderates and spur them towards extremism. Similar patterns arise when sect-sizes are symmetric, but available resources for the two sects are unequal. In this case, while the radical faction of the more resourceful group largely engages in internal indoctrination, radicals from the less resourceful group typically become more violent while their ranks decrease in relative size. These results indicate that when resources are limited the best strategy is to attack rather than internally indoctrinate. Here, retaliatory action from the opposite sect is limited compared with the case of asymmetric sect sizes, since now the groups are equal in size, and the more resourceful one is largely focused on internal proselytizing rather than on attacking. Finally, we allowed for a series of optimal updates where each sect was able to adjust their strategies in response to their opponent’s choices in an alternating fashion. Depending on parameter choices and initial conditions, limit cycles may be observed, although the same general trends described above for Nash equilibria hold: one of the two sects will preferentially engage in internal indoctrination, the other in violently attacking their opponent.

One of the possible ingredients missing from this work is the intervention of third parties to pacify conflict. Examples of such parties could be more powerful groups, nations, or international bodies. For example, one could include a penalty term in the utility function so that large radical factions or large attack rates are more costly due to the imposition of third-party sanctions. We could also relax the allegiance to a specific sect and allow for recruitment of both radicals and moderates to occur across sect lines, for example, through free or coerced religious conversion. In this context, sect populations would no longer be fixed. As presented in our work, attacks do not lead to casualties; allowing for victims could be another mechanism whereby both violence and indoctrination could lead to the possible extinction of a sect. Finally, we could consider multiple interacting sects marked by shifting allegiances, as motivated by the complex dynamics in the Middle East, with ad hoc functional forms for radicalization and de-radicalization, and including possible calibration of parameters through data.

One final observation is that in presenting this model, we have used the general framework of religious and/or ethnic conflict. Our work, however, can also be mapped onto western political debates, given the proper identifications and modifications are introduced. A most notable case is that of two-party democracies where each is characterized by a more radical, active group and by a more moderate, centrist wing. In this case, the attack rates of our model represent the negative, incendiary, statements used by radicals against the opponent party, while internal indoctrination rates can model positive advertisement used by radicals to internally mobilize more moderate supporters. As in our current work, resources are fixed so that a choice must be made on whether to concentrate on negative attacks or on positive statements. We leave the fine tuning of this mapping for future work.

## Data accessibility

This article has no additional data.

## References

[1] Gentile V 2013 *From identity-conflict to civil society restoring human dignity and pluralism in deeply divided societies*. Rome, Italy: LUISS University Press.

[2] McGarry J, O’Leary B 2013 *The politics of ethnic conflict regulation*. New York, NY: Routledge.

[3] Smith D 2016 *SIPRI yearbook 2016: armaments, disarmament, and international security*. Oxford, UK: Oxford University Press.

[4] Gonzalez N 2009 *The Sunni-Shia conflict: understanding sectarian violence in the Middle East*. Mission Viejo, CA: Northia Press.

[5] Taylor M, Horgan J 2006 A conceptual framework for addressing psychological process in the development of the terrorist. *Terror. Polit. Violence* 18, 585–601. (doi:10.1080/09546550600897413)

[6] Horgan J 2008 From profiles to pathways and roots to routes: perspectives from psychology on radicalization into terrorism. *Ann. Am. Acad. Polit. Sov. Sci.* 618, 80–94. (doi:10.1177/0002716208317539)

[7] McCauley C, Moskalenko S 2008 Mechanisms of political radicalization: pathways toward terrorism. *Terror. Polit. Violence* 20, 415–433. (doi:10.1080/09546550802073367)

[8] Castillo-Chávez C, Song B 2003 Models for the transmission dynamics of fanatics. In *Bioterrorism: mathematical modeling applications in homeland security*. Philadelphia PA: SIAM.

[9] Marvel SA, Hong H, Papush A, Strogatz SH 2012 Encouraging moderation: clues from a simple model of ideological conflict. *Phys. Rev. Lett.* 109, 118702 (doi:10.1103/PhysRevLett.109.118702) 23005690 10.1103/PhysRevLett.109.118702

[10] Stauffer D, Sahimi M 2006 Discrete simulation of the dynamics of spread of extreme opinions in a society. *Phys. A* 364, 537–543. (doi:10.1016/j.physa.2005.08.040)

[11] Stauffer D, Sahimi M 2007 Can a few fanatics influence the opinion of a large segment of a society? *Eur. Phys. J. B* 57, 147–152. (doi:10.1140/epjb/e2007-00106-7)

[12] Hegemann R, Smith L, Barbaro A, Bertozzi A, Reid S, Tita G 2011 Geographical influences of an emerging network of gang rivalries. *Phys. A.* 390, 3894–3914. (doi:10.1016/j.physa.2011.05.040)

[13] Berestycki H, Nadal JP, Rodriguez N 2015 A model of riots dynamics: shocks, diffusion and thresholds. *Netw. Heterog. Media* 3, 443–475. (doi:10.3934/nhm.2015.10.443)

[14] Ehrhardt M, Peco M, Tarazona AC, Villanueva RJ, Villanueva-Oller J 2014 Popular support to terrorist organizations: a short-term prediction based on a dynamic model applied to a real case. In *Mathematical modeling in social sciences and engineering*. Hauppauge, NY: Nova Science.

[15] Blank L, Enomoto CE, Gegax D, McGuckin T, Simmons C 2008 A dynamic model of insurgency: the case of the war in Iraq. *Peace Econ. Peace Sci. Publ. Policy* 14, 1–26. (doi:10.2202/1554-8597.1120)

[16] Farley JD 2007 Evolutionary dynamics of the insurgency in Iraq: a mathematical model of the battle for hearts and minds. *Stud. Confl. Terror.* 30, 947–962. (doi:10.1080/10576100701611304)

[17] Chuang YL, D’Orsogna MR, Chou T 2016 A bistable belief dynamics model for radicalization within sectarian conflict. *Q. Appl. Math.* 75, 19–37. (doi:10.1090/qam/1446)

[18] Weidmann NB, Salehyan I 2013 Violence and ethnic segregation: a computational model applied to Baghdad. *Int. Stud. Quart.* 57, 52–64. (doi:10.1111/isqu.12059)

[19] Kruglanski AW, Fishman S 2009 Psychological factors in terrorism and counterterrorism: individual, group, and organizational levels of analysis. *Soc. Issues Policy Rev.* 3, 1–44. (doi:10.1111/j.1751-2409.2009.01009.x)

[20] Borum R 2012 Radicalization into violent extremism. I: a review of social science theories. *J. Strat. Security* 4, 7–36. (doi:10.5038/1944-0472.4.4.1)

[21] Kruglanski AW, Gelfand MJ, Belanger JJ, Sheveland A, Hetiarachchi A, Gunaratna R 2014 The Psychology of radicalization and deradicalization: how significance quest impacts violent extremism. *Polit. Psychol.* 35, 69–93. (doi:10.1111/pops.12163)

[22] Wadhwa P, Bhatia MPS 2015 Measuring radicalization in online social networks using Markov chains. *J. Appl. Secur. Res.* 10, 23–47. (doi:10.1080/19361610.2015.972265)

[23] Bovenkerk F 2011 On leaving criminal organizations. *Crime Law Soc. Change* 55, 261–276. (doi:10.1007/s10611-011-9281-x)

[24] Bjorgo T, Horgan J 2008 *Leaving terrorism behind: individual and collective disengagement*. New York, NY: Routledge.

[25] Crozier B 1960 *The rebels: a study of post war insurrections*. Boston, MA: Beacon Press.

[26] Kfirm I 2014 Sectarian violence and social group identity in Pakistan. *Stud. Confl. Terror.* 37, 457–472. (doi:10.1080/1057610X.2014.903374)

[27] Strogatz SH 2015 *Nonlinear dynamics and chaos: with applications to physics, biology, chemistry, and engineering*, 2nd edn Boulder, CO: Westview Press.

[28] Abrahamian E 2009 Mass protests in the Islamic revolution, 1977–79. In *Civil resistance and power politics: the experience of non-violent action from Gandhi to the present* (eds A Roberts, T Garton Ash), pp. 162–178. Oxford, UK: Oxford University Press.

[29] Von Stackelberg H 2011 *Market structure and equilibrium*. Berlin, Germany: Springer.

[30] Jovanovic B, Rosenthal RW 1988 Anonymous sequential games. *J. Math. Econ.* 17, 77–87. (doi:10.1016/0304-4068(88)90029-8)

[31] Lasry JM, Lions PL 2006 Jeux à champ moyen. I–le cas stationnaire. *C. R. Math.* 343, 619–625. (doi:10.1016/j.crma.2006.09.019)

[32] Lasry JM, Lions PL 2006 Jeux à champ moyen. II–horizon fini et contrôle optimal. *C. R. Math.* 343, 679–684. (doi:10.1016/j.crma.2006.09.018)

[33] Huang M, Malhamé RP, Caines PE 2006 Large population stochastic dynamic games: closed-loop McKean-Vlasov systems and the Nash certainty equivalence principle. *Commun. Infor. Syst.* 6, 221–252. (doi:10.4310/CIS.2006.v6.n2.a2)

